# Molybdenum-Induced Oxidative and Inflammatory Injury and Metabolic Pathway Disruption in Goat Pancreas

**DOI:** 10.3390/metabo15080541

**Published:** 2025-08-09

**Authors:** Longfei Li, Yang Ran, Xiaoyun Shen

**Affiliations:** 1 College of Agriculture and Biology, Liaocheng University, Liaocheng 252000, China; 2310150208@stu.lcu.edu.cn; 2 College of Life Science and Agri-Forestry, Southwest University of Science and Technology, Mianyang 621010, China; yangran_007@yeah.net; 3 Rural Revitalization Project Center, Guizhou Department of Agriculture and Rural Affairs, Guiyang 550000, China

**Keywords:** molybdenum, goat pancreas, injury, metabolomics

## Abstract

**Background**: Molybdenum (Mo) is an essential trace element for animals, but too much intake can cause adverse effects. Due to the metabolic characteristics of goats and other ruminants, they are more susceptible to the cumulative effects of Mo toxicity. A high Mo intake can cause multi-organ toxicity in ruminants, but the mechanism of damage to the pancreas is still unclear. The aim of this study was to systematically analyze the key regulatory pathways of pancreatic injury induced by Mo in goats using a metabolomics approach. **Methods**: Twenty male Yudong Black goats (22.34 ± 1.87 kg, six months) were randomly divided into a control group (fed a basal diet) and the Mo group (fed a basal diet supplemented with 50 mg·kg^−1^ Na_2_MoO_4_·2H_2_O). After 60 days of continuous feeding, their pancreatic tissues were collected and the mineral elements, antioxidant capacity, and inflammatory factors were examined. Untargeted metabolomics based on HILIC UHPLC-Q-EXACTIVE MS was used to analyze changes in metabolites. The core regulatory mechanisms were revealed by KEGG enrichment analysis. **Results**: The results demonstrated that goats in the Mo group showed obvious clinical signs, such as lethargy, loss of appetite, and unsteady gait. The pancreatic tissue of goats in the Mo group exhibited significantly elevated levels of Mo and copper, accompanied by a marked reduction in antioxidant capacity and concurrent increases in inflammatory cytokine levels. Between the Mo group and control group, 167 differentially expressed metabolites were identified. KEGG enrichment analysis showed that it disrupted multiple metabolic pathways, including glycine, serine, and threonine metabolism, cysteine and methionine metabolism, and butanoate metabolism. **Conclusions**: This study mainly revealed, at the metabolomics level, that Mo exposure would disrupt the metabolic pathways related to antioxidant capacity in goat pancreata. It provides new insights into the molecular mechanisms of Mo-induced pancreatic injury in goats.

## 1. Introduction

Molybdenum (Mo), a vital trace element in animals, is involved in the active center formation of xanthine oxidase, sulfite oxidase, and other key enzymes, which is crucial for antioxidant defense and energy metabolism [1,2]. However, industrial activities (such as mining and smelting) and agricultural practices have led to substantial environmental accumulation of Mo. Especially in regions like Jiangxi Province, China, the extraction of tungsten ores has resulted in the discharge of massive Mo-containing tailings. Through long-term accumulation, these tailings have polluted large areas of surrounding water bodies, land, and vegetation, threatening the health of animals and humans [3]. The Mo content may exceed 100 mg·kg^−1^ in contaminated soils, which is nearly 100 times that of normal soil [4]. When animals are exposed to environments with a high Mo content, acute or chronic Mo poisoning can occur, clinically manifested as slow growth, persistent diarrhea, and hair loss [5,6]. It is worth noting that in goats, a typical ruminant, Mo forms thiopolybdate with sulfur (S) in the rumen and creates a complex with copper (Cu), which hinders Cu absorption [7,8]. It is precisely because of the metabolic mode of Mo in the rumen that ruminants such as goats are more sensitive to Mo than monogastric animals and more susceptible to the cumulative effects of Mo toxicity [9,10]. Although it has been confirmed that exposure to high levels of Mo can induce oxidative stress in the liver and inflammation in the kidney [11,12,13], there are still relatively few studies on its mechanism of pancreatic injury [14].

Recent studies found that if (NH_4_)_6_Mo_7_O_24_·4H_2_O was used as a Mo source and a high concentration of Mo was fed to Boer goats for 50 consecutive days, it would lead to decreased appetite, weight loss, diarrhea, and alopecia, as well as oxidative damage, swelling and necrosis of liver cells, and a significant reduction in Cu content in serum. By hindering the absorption of Cu by goats, it causes Cu deficiency [15]. It can also cause anemia in goats, reduce the antioxidant capacity of kidney mitochondria and erythrocyte membranes, and cause pathological damage to tissues and organs [16]. In addition, a high concentration of Mo can significantly reduce the total number and vitality of ciliates in rumen fluid, disrupt the balance among rumen microorganisms, and affect the absorption and utilization of nutrients by ruminants [17,18]. However, traditional toxicological studies are mostly limited to histopathological observation and single biomarker detection, making it difficult to systematically analyze the molecular mechanism of pancreatic injury caused by high Mo content. Compared to other omics technologies (such as transcriptomics or proteomics), metabolomics offers unique advantages for this study. It directly captures downstream metabolic perturbations that reflect functional consequences of molecular changes, bridging the gap between gene or protein expression and physiological phenotypes [19]. This is critical for elucidating toxic mechanisms, as metabolic shifts are often the most sensitive and immediate responses to Mo-induced cellular stress. By profiling small-molecule metabolites and their pathway perturbations, metabolomics provides a holistic perspective on functional alterations, rather than just potential regulatory changes (such as in mRNA or protein levels) [20]. In recent years, this approach has been successfully applied to investigate the toxicity mechanisms of heavy metals such as cadmium and arsenic [21,22]. Nevertheless, its application in Mo toxicity research remains in its initial phase. As the core organ for the synthesis and secretion of digestive enzymes such as amylase, lipase, and trypsin, the dysfunction of the pancreas will directly lead to the disorders of nutrient metabolism and impact the body’s normal growth and development [23]. Given the critical role of the pancreas in digestive and absorptive functions in animals, investigating the effects of Mo accumulation on the pancreas is of paramount importance.

This study mainly utilized metabolomics analysis to elucidate the related metabolic pathway changes underlying Mo excess-induced pancreatic injury in goats. By screening for differentially expressed metabolites (DEMs) and identifying key metabolic pathways, we aimed to uncover novel molecular evidence for Mo toxicity, thus providing new ideas for investigating the molecular mechanisms of Mo-induced pancreatic injury in goats.

## 2. Materials and Methods

### 2.1. Managing Animals and Preparing Samples

Twenty healthy 6-month-old male Yudong Black goats (body weight: 22.34 ± 1.87 kg) were selected from a goat farm in Chongqing, China. All experimental goats were vaccinated according to the farm’s health management protocol, including goat pox, foot-and-mouth disease, immunizations against peste des petits ruminants, and clostridial vaccine. All vaccinations were maintained within the valid immunization window. Additionally, deworming treatment was administered prior to the trial. The experimental animals were housed in semi-open sheep pens with wooden slatted floors. The sheep pens were thoroughly disinfected in advance using disinfectant solutions. All goats had free access to water and were fed at 08:00 and 17:00, respectively, every day.

Two groups of ten goats each were randomly selected from twenty goats. The control group (CON group) was provided with the basal diet (Table S1). Additionally, the Mo group’s basic food was supplemented with 50 mg·kg^−1^ Na_2_MoO_4_·2H_2_O (Shanghai Maclin Biochemical Technology Co., Ltd., Shanghai, China). The Mo concentration used was chosen based on previous research. A Mo concentration of 50 mg·kg^−1^ in the diet has been demonstrated to induce adverse effects in ruminants, such as splenic cell apoptosis and muscle tissue damage [16,21]. The treatment period lasted sixty days.

During the experiment, the clinical signs of different groups of goats were observed and recorded every 15 days. When the experiment was finished, all goats were fasted for 24 h and forbidden to drink water for 2 h and then euthanized. The abdominal cavity was immediately opened, and the internal organs were removed. The pancreas was carefully stripped of fatty and connective tissue on an ice tray, then wrapped in foil, and numbered. It was then promptly frozen in liquid nitrogen. After sampling, all samples were transferred to −20 °C and frozen for use.

Ten appropriate samples of goat pancreas tissue were taken from the Mo and CON groups, respectively, and dried at 105 °C until constant weight. Subsequently, 0.20 g of the dried sample was weighed and placed into a digestion tube. Then, 3.0 mL of 65% nitric acid, 2.0 mL of 30% hydrogen peroxide, and 3.0 mL of ultrapure water were added sequentially. The mixture was thoroughly mixed, allowed to stand for 10 min, and then processed using an all-round microwave digestion instrument (TOPEX, PreeKem, Shanghai, China), the microwave digestion program is shown in Table S2. The resulting digestion solution was quantitatively transferred to a 100 mL volumetric flask and diluted to volume with ultrapure water, preparing it for subsequent mineral element analysis.

Another appropriate amount of pancreatic tissue was thawed at 4 °C. When the sample was nearly fully thawed, 0.50 g of the sample was weighed. Then, 4.5 mL of 0.9% saline was added, and the mixture was processed using a high-speed tissue homogenizer (KZ-III-F, Servicebio, Mianyang, China) in an ice-water bath to prepare a 10% homogenate. The homogenate was then centrifuged at 3000 rpm·min^−1^ for 10 min at 4 °C. The supernatant was collected and prepared for analysis of antioxidant indicators and inflammatory factors.

### 2.2. Determination of Mineral Elements in Goat Pancreas

Single-element standard solutions (1000 μg/mL) were pipetted, and each was quantitatively diluted with 5% nitric acid to prepare a solution with a concentration of 200 μg/L. The diluted solution was then quantitatively diluted with 2% nitric acid to prepare solutions with a series of concentrations: 1, 5, 10, 50, 100, and 200 μg/L. The optimal wavelength for each element was selected, and the above-prepared standard solutions were sequentially detected using an inductively coupled plasma optical emission spectrometer (ICP-OES OPTIMA 8000, PerkinElmer, Shelton, CT, USA) to plot the standard curve and determine the detection limit. The processed samples were sequentially analyzed on the instrument, and the content of each element, including Mo, iron (Fe), Cu, and zinc (Zn), was calculated according to the standard curve.

### 2.3. Detection of Antioxidant Indexes and Inflammatory Factors in Goat Pancreas

Five antioxidant indexes, including superoxide dismutase (SOD), catalase (CAT), total antioxidant capacity (T-AOC), glutathione peroxidase (GSH-Px) and malondialdehyde (MDA), and four inflammatory factors, containing interleukin-2 (IL-2), interleukin-6 (IL-6), interleukin-10 (IL-10) and tumor necrosis factor-α (TNF-α), were detected according to the instructions of ELISA diagnostic kit (Nanjing Jiancheng Bioengineering Research Institute Co., LTD, Nanjing, China). Technical parameters of different ELISA diagnostic kits are shown in Table S3.

### 2.4. Metabolomics Analysis

Six pancreatic tissue samples were selected from each of the Mo group and CON group for metabolomics analysis. A 50 mg sample was weighed, and 400 μL of pre-cooled acetonitrile/methanol/water solution (volume ratio = 4:4:2) was added. After mixing thoroughly, the mixture was allowed to stand at −20 °C for 60 min. Subsequently, centrifugation was performed at 14,000 g and 4 °C for 20 min. The supernatant was collected and subjected to vacuum drying. For mass spectrometry analysis, 100 μL of acetonitrile/water solution (volume ratio = 1:1) was added for reconstitution. After mixing well, centrifugation was carried out at 14,000 g and 4 °C for 15 min, and 2 μL of the supernatant was taken for LC-MS/MS analysis. To track and assess the system’s stability and the accuracy of the experimental data, the quality control (QC) samples were added to the sample analysis queue. Each sample was detected in both positive and negative ion modes using electrospray ionization. After separation by UHPLC, the samples were analyzed by a Thermo QE HF-X mass spectrometer. The data collected by mass spectrometry were preprocessed to output a three-dimensional data matrix. The identification of metabolite structures was performed by means of accurate mass number matching (<25 ppm) and secondary spectrum matching, with searches conducted in the databases such as HMDB, Massbank, and KEGG. Pattern recognition was performed using SIMCA-P 14.1 (Umetrics, Umea, Sweden). After the data were preprocessed by Pareto-scaling, multivariate statistical analyses were carried out, including principal component analysis (PCA), partial least squares discriminant analysis (PLS-DA), and orthogonal partial least squares discriminant analysis (OPLS-DA). Univariate statistical analyses included Student’s *t*-test and fold change analysis [21].

### 2.5. Data Analysis

Prior to statistical analysis, normality was assessed using the Shapiro–Wilk test, and homogeneity of variance was evaluated via Levene’s test. For comparisons between the two groups, the significant differences in the experimental results were analyzed through the independent samples of *t*-test in SPSS 23.0 software (Statistical Package for the Social Sciences, version 23.0, Inc., Chicago, IL, USA) when data met the assumptions of normality and variance homogeneity. Graphs were drawn by GraphPad Prism (version 9.0.0, San Diego, CA, USA).

## 3. Results

### 3.1. Clinical Signs of Goats

On day 15 of the experiment, compared with the CON group, goats in the Mo group exhibited obvious clinical signs of decreased feed intake. By day 30, clinical signs such as diarrhea, unsteady gait, and lethargy were observed. With the prolongation of experimental treatment time, the clinical signs of goats became increasingly severe.

### 3.2. Changes in Mineral Elements in Goat Pancreas

**Figure 1:**
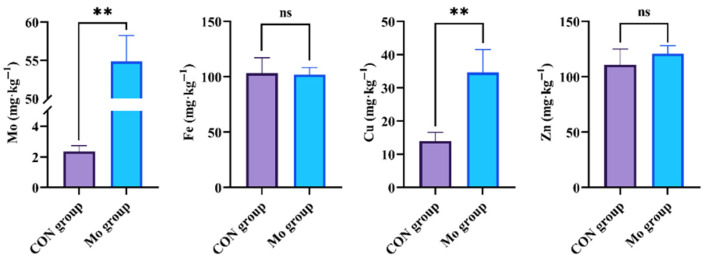
Changes of high Mo content on mineral elements in goat pancreas. ** denotes statistical significance at *p* < 0.01, whereas ns denotes no statistical significance at *p* > 0.05.

The Mo group’s goat pancreas had significantly higher levels of Mo and Cu than the CON group’s (*p* < 0.01). However, the Fe and Zn levels did not change significantly (*p* > 0.05) (Figure 1).

### 3.3. Effects of Antioxidant Capacity in Goat Pancreas

**Figure 2:**
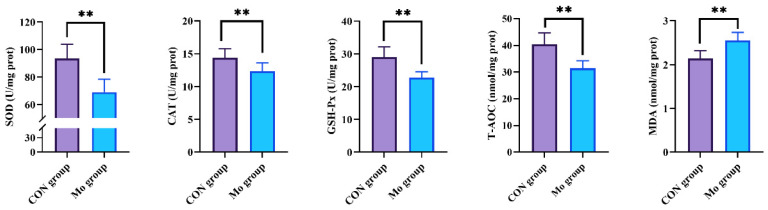
Effects of high Mo content on antioxidant properties of goat pancreas. ** denotes statistical significance at *p* < 0.01.

The Mo group had significantly lower levels of SOD, CAT, GSH-Px, and T-AOC than the CON group (*p* < 0.01), as seen in Figure 2, and a significantly greater MDA content than the CON group (*p* < 0.01).

### 3.4. Influences of Inflammatory Factor in Goat Pancreas

**Figure 3:**
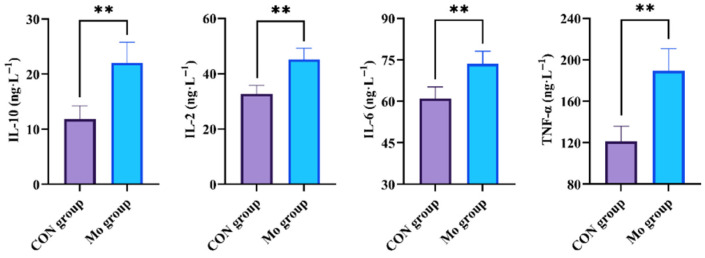
Influences of high Mo content on inflammatory factor in goat pancreas. ** denotes statistical significance at *p* < 0.01.

Figure 3 revealed that the levels of TNF-α, IL-10, IL-6, and IL-2 in goat pancreas of the Mo group were significantly higher than those in the CON group (*p* < 0.01).

### 3.5. Quality Validation of Metabolomics Data

The complete spectrum analysis of the samples in this study was conducted using the HILIC UHPLC-Q-EXACTIVE MS technology in conjunction with a data-dependent acquisition approach. Before pancreas sample analysis, the system stability of instruments and equipment was checked by comparing QC sample spectra with PCA. The total ion chromatograms (TIC) of samples from QC, Mo group, and CON group were overlapped and compared, respectively, in positive and negative ion modes. The findings indicated that the response intensity of each chromatographic peak and the retention time of the ion peak of QC samples basically coincided (Figure S1A,B). In positive ion mode, 73.40% of the metabolites in the samples had a relative standard deviation (RSD) of ≤30% in the QC samples. In negative ion mode, 70.37% of the metabolites in the samples showed an RSD of ≤30% in the QC samples. This indicated that the error caused by the instrument was small in the sample analysis process, and the method had good stability and repeatability. The trend of TIC charts of the Mo group and CON group was roughly the same, respectively (Figure S1C–F), proving that the information was trustworthy and suitable for analysis.

### 3.6. Results of Supervised Multivariate Statistical Analysis

**Figure 4:**
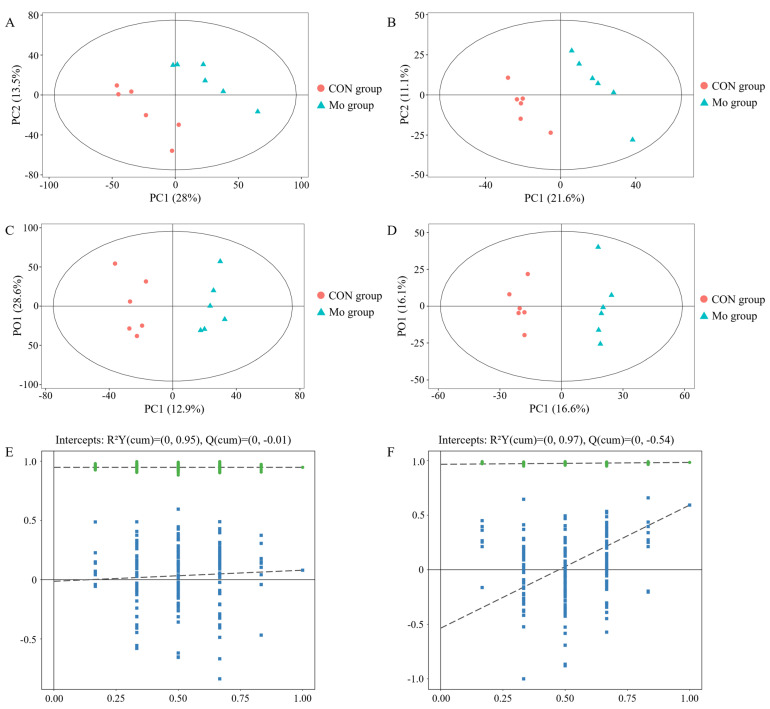
Results of supervised multivariate statistical analysis. (**A**,**B**) PLS-DA scores and replacement test of pancreatic samples. (**C**,**D**) OPLS-DA score diagram of samples. (**E**,**F**) OPLS-DA replacement test. The graph on the left showed the positive ion mode, and the one on the right showed the negative ion mode.

To further identify ion peaks that could be used to distinguish between Mo group and CON groups, a supervised PLS-DA model was created. According to Figure 4A,B, PLS-DA scores showed that the separation trend of Mo group and CON group was obvious in positive and negative ion modes, respectively, indicating that high Mo treatment caused changes in the levels of some metabolites in goat pancreas.

The modification of OPLS-DA on the basis of PLS-DA could further enhance the model’s efficacy and capacity for analysis. A significant trend of separation between samples in the Mo group and CON group was evident in the OPLS-DA score plot (Figure 4C–F), indicating differences in metabolite levels between different groups. In this research, R2 and Q2 in positive and negative ion modes were ≥0.5, and the model was stable and reliable. The Q2 intercept of the displacement test diagram of the OPLS-DA model was less than zero in both positive and negative ion modes, indicating that the OPLS-DA model did not overfit.

### 3.7. Analysis of DEMs

**Figure 5:**
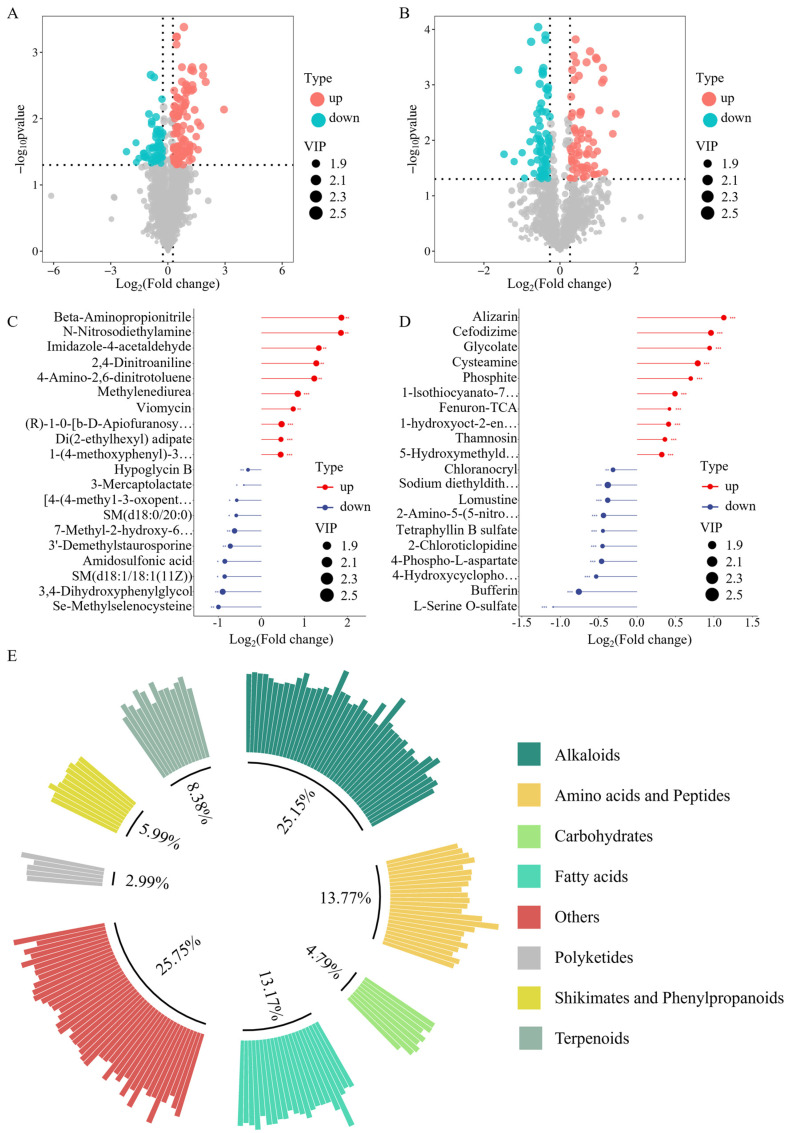
Identification and screening of DEMs. (**A**,**B**) Volcanic map of pancreatic DEMs screening. (**C**,**D**) The top 10 DEMs with the smallest *p* values among the up-regulated and down-regulated metabolites. The negative ion mode was displayed on the right graph, while the positive ion mode was displayed on the left. Red represented an increase and blue represented a decrease. (**E**) Classification of the screened DEMs.

The criteria for screening DEMs were *p* < 0.05 and FC < 0.8 or > 1.2. In the positive ion mode, 111 up-regulated DEMs and 56 down-regulated DEMs were screened. In the negative ion mode, 73 up-regulated DEMs and 69 down-regulated DEMs were screened (Figure 5A,B). To better observe the differences, ten DEMs with the smallest *p* values from the up-regulation group and the down-regulation group were selected and a lollipop graph was created (Figure 5C,D). Detailed information on the top 30% of significantly up-regulated and down-regulated DEMs was provided in Table S4. The results of cluster analysis showed that the Mo group was closely clustered, and the CON group was separated (Figure S2). The screened DEMs were classified into alkaloids, amino acids and peptides, carbohydrates, fatty acids, polyketides, shikimates and phenylpropanoids, terpenoids, and other categories (Figure 5E).

### 3.8. KEGG Metabolic Pathway Analysis of DEMs

**Figure 6:**
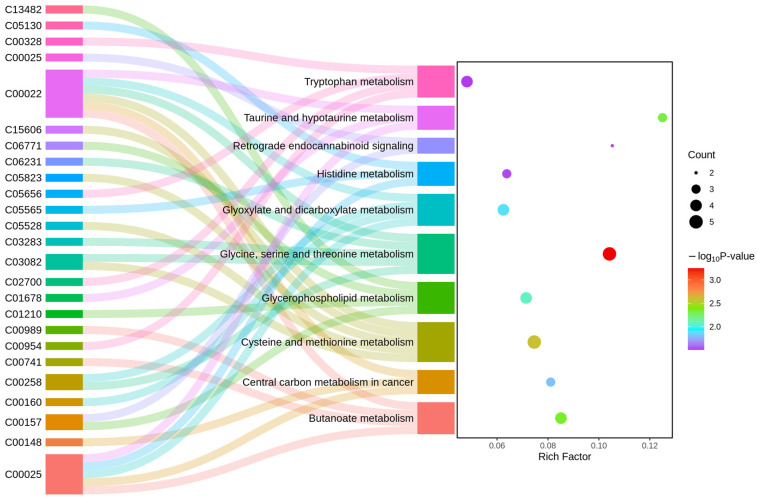
KEGG pathway analysis of pancreatic DEMs.

A total of 10 metabolic pathways were found to be significantly enriched (*p* < 0.05) by KEGG metabolic pathway analysis of DEMs. They were glycine, serine and threonine metabolism, cysteine and methionine metabolism, butanoate metabolism, taurine and hypotaurine metabolism, glycerophospholipid metabolism, glyoxylate and dicarboxylate metabolism, central carbon metabolism in cancer, histidine metabolism, retrograde endocannabinoid signaling, and tryptophan metabolism (Figure 6, Table S5).

## 4. Discussion

In the present study, goats exhibited obvious clinical signs such as reduced feed intake, diarrhea, unsteady standing, and lethargy after the continuous intake of excessive Mo for a certain period. This is very similar to the manifestations of Mo poisoning in cattle, including gaseous diarrhea and lameness [24,25]. Excessive Mo intake in animals induces a significant increase in blood Mo levels alongside a marked reduction in Cu concentrations, which occurs in cattle as well [26,27]. Intriguingly, our results revealed concurrent elevations of both Mo and Cu in pancreatic tissues, and it aligned with the findings of Ryssen et al. [28]. This may be potentially attributed to erythrocyte rupture triggered by Mo toxicity, which facilitates Cu leakage and subsequent deposition in visceral organs such as the pancreas [26].

In the Mo group, the pancreatic tissues of goats exhibited significantly reduced activities of antioxidant enzymes (SOD, CAT, and GSH-Px) and elevated MDA levels, indicating oxidative stress and compromised antioxidant defense mechanisms. The antioxidant system in animals constitutes a complex network designed to protect cells from free radical damage, comprising enzymatic and non-enzymatic components [29]. The system of enzymes consists of key antioxidants such as GSH-Px, which scavenge free radicals via catalytic reactions to maintain cellular integrity and function [30]. The non-enzymatic system encompasses antioxidants (such as vitamin C and cysteine) and trace elements (including Cu, Fe, and Zn), synergistically counteracting oxidative damage [31]. Dysregulation of this system leads to excessive free radical accumulation, exacerbating oxidative injury [32]. Hydrogen peroxide is produced by SOD from superoxide radicals, and CAT and GSH-Px then detoxify the hydrogen peroxide [33]. GSH-Px reduces lipid hydroperoxides to corresponding alcohols and converts free H_2_O_2_ to water [34]. MDA, a hallmark product of lipid peroxidation, serves as a sensitive indicator of oxidative damage, with elevated levels reflecting severe lipid oxidation [35]. The integrated efficacy of the antioxidant system, as reflected by T-AOC, represents both the compensatory potential against external stressors and the ability to neutralize free radicals. Declines in T-AOC signify diminished antioxidant capacity, heightening disease susceptibility [36]. Ren et al. observed chronic selenium toxicity in Przewalski’s gazelles inhabiting selenium-rich regions near Qinghai Lake, manifesting as anemia and reduced antioxidant activity [37]. Similarly, Solaiman et al. demonstrated that excessive dietary (NH_4_)_2_MnO_4_ in Boer goats decreased hepatic Cu and Fe levels while suppressing immune function [38]. In this paper, the pancreas, a vital endocrine and digestive organ, displayed heightened sensitivity to oxidative stress. The elevated oxidative stress induced by high Mo exposure likely disrupts pancreatic cellular function, impairing digestive enzyme activity and ultimately jeopardizing overall animal health.

The observed upregulation of pancreatic TNF-α, IL-6, IL-2, and IL-10 in goats with excessive Mo intake reflected a multifaceted inflammatory interplay. When cells subjected to oxidative damage are cleared by the body, it triggers the release of cytokines. The increase in cytokines activates more inflammatory cells, further exacerbating the inflammatory response [39]. TNF-α, a pivotal pro-inflammatory cytokine, drives neutrophil infiltration and amplifies tissue damage via apoptosis and oxidative bursts, while IL-6 exhibits dual roles as both an acute-phase mediator and a promoter of fibrotic remodeling [40]. IL-2, a Th1 immunity marker, suggests T-cell activation against Mo-induced cellular stress, whereas IL-10 acts as a critical immunosuppressive regulator to constrain excessive inflammation [41]. The concurrent elevation of these mediators implied a dynamic equilibrium between pro-inflammatory injury and compensatory repair mechanisms. Under excessive Mo intake conditions, the elevated levels of TNF-α, IL-6, and IL-2 exacerbated pancreatic injury, while the increased IL-10 content may reflect a compensatory mechanism to mitigate inflammatory damage. However, as pancreatic samples were only collected at the end of the experiment, longitudinal sampling of experimental animals was lacking, making it impossible to monitor the dynamic changes in various indicators. In future studies, non-lethal sampling methods are expected to be used to validate these findings.

The PLS-DA and OPLS-DA analyses revealed significant differences in endogenous metabolites within the pancreatic tissues of goats between the Mo group and CON group. DEMs were mapped to the KEGG database to identify associated metabolic pathways. A total of 167 DEMs were identified in positive ion mode, while 142 DEMs were detected in negative ion mode. Functioning as substrates or intermediates, these metabolites participate in diverse physiological processes. Notably, 10 metabolic pathways were significantly enriched, including glycine, serine, and threonine metabolism, cysteine and methionine metabolism, butanoate metabolism, taurine and hypotaurine metabolism, glycerophospholipid metabolism, glyoxylate and dicarboxylate metabolism, central carbon metabolism in cancer, histidine metabolism, retrograde endocannabinoid signaling, and tryptophan metabolism.

Dysregulation of the glycine, serine, and threonine metabolic pathway impairs the biosynthesis of serine and glycine. As critical precursors for glutathione, a key intracellular antioxidant, reduced availability of serine and glycine subsequently diminishes glutathione synthesis [42,43]. This reduction in glutathione compromises the organism’s capacity to scavenge reactive oxygen species (ROS) when intracellular ROS levels rise, thereby exacerbating oxidative damage.

In protein structures, all amino acid residues are susceptible to oxidation by various forms of ROS, with methionine residues being particularly vulnerable as they are sensitive to almost all types of ROS [44]. Methionine residues tend to react with ROS, converting into methionine sulfoxide, thereby inactivating ROS. Eventually, methionine sulfoxide is reduced back to methionine by thioredoxin under enzymatic catalysis, forming a methionine redox cycle. Each cycle can eliminate harmful substances such as excessive ROS and lipid peroxides [45]. Marchetti et al. used targeted gene silencing to downregulate the expression levels of key enzymes in this cycle, resulting in varying degrees of inhibition of the cycle. This led to the accumulation of ROS in human lens cells and even cell death induced by oxidative stress [46]. Yermolaieva et al. employed adenovirus-mediated overexpression of key enzymes in the cycle, which increased the frequency of the methionine redox cycle. They found that this could significantly reduce ROS production in PC12 cells under hypoxic conditions and alleviate the degree of oxidative damage [47]. Similarly to methionine residues, cysteine residues are also easily oxidized. Their unique chemical properties enable them to readily react with H_2_O_2_, thus conferring redox regulatory properties [48]. Additionally, as a precursor of glutathione, cysteine is the rate-limiting amino acid for glutathione synthesis in the transsulfuration pathway. A higher cysteine content corresponds to a faster rate of glutathione synthesis [49]. Badaloo et al. found that cysteine supplementation in humans could accelerate the synthesis rate of glutathione and increase its concentration, thereby enhancing the body’s antioxidant capacity [50]. Furthermore, Yin et al. demonstrated that dietary supplementation of cysteine in mice could significantly increase the concentration of glutathione in the liver and improve antioxidant capacity [51]. In the present study, disturbances in cysteine and methionine metabolic pathways lead to a reduction in the synthesis of cysteine and methionine. This results in a decreased frequency of the methionine redox cycle and a slowed rate of glutathione synthesis. Consequently, ROS that should normally be scavenged by the methionine redox cycle and glutathione begin to accumulate in large quantities, leading to a significant reduction in the body’s antioxidant capacity.

Butyrate, a short-chain fatty acid, can be obtained from dietary intake or produced through gut microbial fermentation of indigestible fibers [52]. Once generated, it is rapidly absorbed in the intestine and serves as both an energy source and a signaling molecule in diverse cell types. Notably, butyrate acts as the primary energy substrate for colonic epithelial cells [53]. Beyond its role in modulating intestinal barrier function, butyrate exhibits anti-inflammatory properties and maintains colonic epithelial homeostasis by regulating gene expression, cell differentiation, and apoptosis. Short-chain fatty acids, including butyrate, inhibit systemic inflammation by suppressing immune cell infiltration into peripheral tissues via reduced chemotaxis and cell adhesion, while significantly lowering pro-inflammatory serum markers such as TNF-α [54]. Additionally, it plays a critical role in weight regulation and glycemic control [55].

Hypotaurine is derived from the metabolic pathways of cysteine degradation and pantothenic acid synthesis. Cysteine is oxidized to cysteine sulfonic acid, which is then decarboxylated to form hypotaurine. Subsequently, hypotaurine is oxidized to taurine under the action of hypotaurine dehydrogenase [56]. Taurine performs multiple functions in cells, particularly exerting a cell-protective role through its antioxidant and anti-inflammatory effects. It neutralizes hypochlorous acid by forming taurine chloramine, thereby reducing the production of ROS [57]. Moreover, taurine can improve the expression of nicotinamide adenine dinucleotide ubiquinone oxidoreductase by binding to tRNA, which helps alleviate oxidative stress-induced damage [58]. When the taurine and hypotaurine metabolic pathways are disrupted, the normal physiological functions of taurine are impaired. This leads to an increase in ROS, exacerbation of oxidative stress damage, and the induction of a certain degree of inflammatory response.

Glycerophospholipids, the predominant lipids in mammalian cell membranes, not only constitute the lipid bilayer but also regulate critical cellular functions such as transport processes and protein activity. They are essential components of lipoproteins, influencing their metabolic dynamics and functionality [59]. The dysregulation of glycerophospholipid metabolism may alter membrane composition and fluidity, impairing membrane protein function and subsequently disrupting cellular transport and signal transduction.

Histidine, a basic amino acid with an imidazole side chain, is one of the least abundant amino acids in human proteins. Its metabolism diverges into multiple pathways: methylation to form 1-methylhistidine or 3-methylhistidine; transamination to imidazole-pyruvate, followed by reduction to imidazole-lactate; decarboxylation to histamine; or non-oxidative deamination catalyzed by histidine ammonia-lyase to produce urocanic acid and ammonia [60]. The unique imidazole ring of histidine confers distinct chemical properties, including proton buffering and antioxidant activity. These functions involve free histidine, histidine-containing peptides, and histidine residues in proteins [61]. Histidine-rich glycoproteins in vertebrate plasma interact with ligands such as zinc and play pivotal roles in immunity. For instance, heavy metal ions (such as Cu and Zn) induce neurotoxicity, which can be attenuated by histidine-mediated detoxification [62]. Thus, perturbations in histidine metabolism caused by excessive Mo may compromise immune cell function, increasing susceptibility to infections and diseases.

## 5. Conclusions

This study revealed the effects of Mo exposure on the goat pancreas at the metabolomic level. Our results indicated that Mo exposure disrupts metabolic pathways associated with antioxidant capacity, leading to a significant reduction in the antioxidant capacity of the pancreas. It also caused imbalances in trace elements, significantly increased the accumulation of Mo and Cu in the pancreas, and induced inflammatory responses. These findings provide new insights into the molecular mechanisms underlying Mo-induced pancreatic damage in goats.

## Data Availability

The datasets generated and analyzed in the current study are available from the corresponding author upon reasonable request. The data are not publicly available due to privacy.

## Supplementary Materials

The following supporting information can be downloaded at: https://www.mdpi.com/article/10.3390/metabo15080541/s1, Table S1: Basic nutritional values and makeup of the diet; Table S2: Microwave digestion program; Table S3: Technical parameters of different ELISA diagnostic kits; Table S4: The top 30% DEMs identified in goat pancreas; Table S5: Significantly enriched KEGG metabolic pathway; Figure S1: Comparison of TIC of each sample; Figure S2: Cluster analysis of DEMs in goat pancreas.

## Abbreviations

The following abbreviations are used in this manuscript:

Mo	Molybdenum
S	Sulfur
Cu	Copper
DEMs	Differentially expressed metabolites
Fe	Iron
Zn	Zinc
SOD	Superoxide dismutase
CAT	Catalase
T-AOC	Total antioxidant capacity
GSH-Px	Glutathione peroxidase
MDA	Malondialdehyde
IL-2	Interleukin-2
IL-6	Interleukin-6
IL-10	Interleukin-10
TNF-α	Tumor necrosis factor-α
QC	Quality control
PCA	Principal component analysis
PLS-DA	Partial least squares discriminant analysis
OPLS-DA	Orthogonal partial least squares discriminant analysis
TIC	Total ion chromatogram
ROS	Reactive oxygen species
