# Supplementary material for: Molybdenum-Induced Oxidative and Inflammatory Injury and Metabolic Pathway Disruption in Goat Pancreas
Source: Metabolites. 2025 Aug 9;15(8):541. doi: 10.3390/metabo15080541 (PMC12388552; doi:10.3390/metabo15080541)
Supplement: Supplementary file 1 [file metabolites-15-00541-s001.zip › metabolites-3711246-supplementary.pdf]

Supplementary

Molybdenum-Induced Oxidative and Inflammatory Injury and Metabolic Pathway Disruption in Goat Pancreas

Longfei Li [1], Yang Ran [2] and Xiaoyun Shen [1,2,3,*]

[1] College of Agriculture and Biology, Liaocheng University, Liaocheng 252000, China; 2310150208@stu.lcu.edu.cn
[2] College of Life Science and Agri-Forestry, Southwest University of Science and Technology, Mianyang 621010, China; yangran_007@yeah.net
[3] Rural Revitalization Project Center, Guizhou Department of Agriculture and Rural Affairs, Guiyang 550000, China
* Correspondence: shenxy@swust.edu.cn

Table S1. Basic nutritional values and makeup of the diet.

Diet ingredient	Content (%)	Nutrient level	Content (%)
Corn grain	28.36	DE ($MJ \cdot kg^{-1}$)	13.65
Corn silage	36.44	CP	14.21
Wheat bran	15.47	ADF	23.47
Soybean meal	6.32	NDF	32.52
Peanut shell	8.68	Ca	0.54
Premix	2.78	P	0.37
NaCl	0.50	-	-
$CaHPO_4$	0.45	-	-
$NaHCO_3$	1.00		
Total	100		

Each kilogram of premix contained 10,000 IU Vitamin A, 32,000 IU Vitamin D, 150 IU Vitamin E, 250 mg niacin, 80 mg pantothenic acid, 5 mg biotin, 700 mg iron, 100 mg copper, 600 mg manganese; 500 mg Zinc, 7.55 mg iodine, 3.55 mg selenium. The DE was calculated and the rest was measured. The Mo content is $0.5 mg \cdot kg^{-1}$.

Table S2. Microwave digestion program.

Program steps	Temperature (°C)	Holding time
1	100	3
2	140	3
3	160	3
4	180	3
5	190	15

Table S3. Technical parameters of different ELISA diagnostic kits.

Product Name	Detection indicators	Assay range	Detection sensitivity	OD wavelength	Instrument	Inter-assay coefficients of variation	Intra-assay coefficients of variation
SOD Detection Kit	SOD	0.2-18 U/mL	0.2 U/mL	450 nm	Microplate reader (Epoch, BioTeK, America)	＜5%	＜3%
CAT Detection Kit	CAT	1-100 U/mL	1 U/mL	405 nm		＜4%	＜6%
GSH-Px Detection Kit (DTNB Method)	GSH-Px	1.25-80 μmol/L	1.25 μmol/L	412 nm		<5%	<3%
MDA Detection Kit	MDA	0.548-100 μmol/L	0.548 μmol/L	532 nm		＜5%	＜5%
IL-2 ELISA Kit	IL-2	9.38-1000 pg/mL	9.38 pg/mL	450 nm		<10%	<10%
IL-6 ELISA Kit	IL-6	0.131-800 pg/mL	0.131 pg/mL	450 nm		<10%	<10%
IL-10 ELISA Kit	IL-10	18.75-2000 pg/mL	18.75 pg/mL	450 nm		<10%	<10%
TNF-alpha ELISA Kit	TNF-α	6.56-500 pg/mL	6.56 pg/mL	450 nm		<10%	<10%

Table S4. The top 30% DEMs identified in goat pancreas.

Ionization mode	Metabolites	Expression change	Fold change	VIP	*P*-value
ESI(+)	Glycolic acid	Up	7.71	2.13	0.007
ESI(+)	Amifostine	Up	3.96	2.30	0.003
ESI(+)	Beta-Aminopropionitrile	Up	3.62	2.37	0.002
ESI(+)	N-Nitrosodiethylamine	Up	3.59	2.35	0.002
ESI(+)	Cucurbitine	Up	3.26	2.07	0.013
ESI(+)	2,5-Dihydro-4,5-dimethyl-2-(2-methylpropyl)thiazole	Up	3.00	1.90	0.029
ESI(+)	Tripropylamine	Up	2.98	2.03	0.011
ESI(+)	1-Deoxy-D-glucitol	Up	2.80	1.93	0.019
ESI(+)	Biphenyl	Up	2.65	2.11	0.007
ESI(+)	Imidazole-4-acetaldehyde	Up	2.52	2.29	0.002
ESI(+)	Imidazolone	Up	2.50	2.30	0.004
ESI(+)	Methylthiouracil	Up	2.50	2.00	0.026
ESI(+)	1-(1-Oxopropyl)-1H-imidazole	Up	2.43	2.31	0.003
ESI(+)	2,4-Dinitroaniline	Up	2.42	2.40	0.002
ESI(+)	Donepezil metabolite M4	Up	2.41	1.83	0.041
ESI(+)	Vinyl chloride	Up	2.36	2.23	0.004
ESI(+)	4-Amino-2,6-dinitrotoluene	Up	2.34	2.39	0.002
ESI(+)	Amidosulfonic acid	Down	0.56	2.10	0.011
ESI(+)	SM(d18:1/18:1(11Z))	Down	0.55	2.00	0.012
ESI(+)	Arginylvaline	Down	0.55	1.68	0.047
ESI(+)	SM(d18:0/12:0)	Down	0.54	1.67	0.042
ESI(+)	3,4-Dihydroxyphenylglycol	Down	0.54	2.37	0.002
ESI(+)	2,4,6-Triaminotoluene	Down	0.51	1.90	0.028
ESI(+)	Alanyl-Leucine; CE10; RDIKFPRVLJLMER-UHFFFAOYSA-N	Down	0.50	1.81	0.036
ESI(+)	Se-Methylselenocysteine	Down	0.50	2.03	0.009
ESI(+)	Pyruvate	Down	0.47	1.79	0.039
ESI(+)	Fructoselysine	Down	0.43	1.86	0.038
ESI(+)	6-{4-[3-(3,7-dimethylocta-2,6-dien-1-yl)-5,7-dihydroxy-6-(4-hydroxy-3-methylbut-2-en-1-yl)-4-oxo-3,4-dihydro-2H-1-benzopyran-2-yl]-3-hydroxyphenoxy}-3,4,5-trihydroxy-oxane-2-carboxylic acid	Down	0.43	1.88	0.037
ESI(+)	Cholesterol glucuronide	Down	0.42	1.89	0.033
ESI(+)	MG(0:0/18:1(11Z)/0:0)	Down	0.41	1.68	0.033
ESI(+)	1,2,3,4-Tetrahydroisoquinoline	Down	0.35	1.61	0.040
ESI(+)	L-Kynurenine	Down	0.32	1.53	0.046
ESI(+)	Methyl linoleate	Down	0.31	1.62	0.023
ESI(+)	Methacholine	Down	0.22	1.80	0.031
ESI(-)	Fensulfothion	Up	2.77	1.92	0.003
ESI(-)	Anagrelide	Up	2.62	1.82	0.008
ESI(-)	3,4,5-trihydroxy-6-[(3-methoxy-3-oxopropanoyl)oxy]oxane-2-carboxylic acid	Up	2.26	1.54	0.037
ESI(-)	Edetic Acid	Up	2.21	2.08	0.001
ESI(-)	Alizarin	Up	2.19	2.16	< 0.001
ESI(-)	Pirinixic acid	Up	2.15	2.05	0.001
ESI(-)	Enflurane	Up	2.06	1.98	0.003
ESI(-)	N(tele)-methylhistaminium	Up	2.02	1.41	0.040
ESI(-)	L-Glutamic acid	Up	1.97	1.67	0.016

ESI(-)	Cefodizime	Up	1.95	2.19	< 0.001
ESI(-)	Methyl levulinate	Up	1.93	1.46	0.041
ESI(-)	Threoninyl-Arginine	Down	0.65	1.77	0.009
ESI(-)	gamma-Chaconine	Down	0.62	1.48	0.040
ESI(-)	Sodium 3-ethyl-7-isopropyl-1-azulenesulfonate	Down	0.61	1.87	0.006
ESI(-)	Bufferin	Down	0.59	2.21	< 0.001
ESI(-)	Cyclopentolate	Down	0.59	1.59	0.025
ESI(-)	alpha-Chlorohydrin	Down	0.58	1.67	0.011
ESI(-)	Alamarine	Down	0.52	1.61	0.048
ESI(-)	LysoPE(0:0/16:0)	Down	0.50	1.64	0.017
ESI(-)	L-Serine O-sulfate	Down	0.47	2.03	0.001
ESI(-)	5-Chloro-6-methoxy-2(3H)-benzoxazolone	Down	0.43	1.58	0.024
ESI(-)	Dibutyl disulfide	Down	0.36	1.75	0.018

Table S5. Significantly enriched KEGG metabolic pathway.

ID	Description	*P*-value	Up Count	Up Gene	Down Count	Down Gene
chx00260	Glycine, serine and threonine metabolism	0.00056453	3	C03283<br>C06231<br>C00258	2	C00022<br>C03082
chx00270	Cysteine and methionine metabolism	0.00258689	1	C05528	4	C00022<br>C15606<br>C03082<br>C05823
chx00650	Butanoate metabolism	0.00443743	1	C00025	3	C00022<br>C00741<br>C00989
chx00430	Taurine and hypotaurine metabolism	0.00470665	2	C00025<br>C01678	1	C00022
chx00564	Glycerophospholipid metabolism	0.00830271	3	C13482<br>C01210<br>C06771	1	C00157
chx00630	Glyoxylate and dicarboxylate metabolism	0.01319315	3	C00025<br>C00160<br>C00258	1	C00022
chx05230	Central carbon metabolism in cancer	0.01583062	1	C00025	2	C00022<br>C00148
chx00340	Histidine metabolism	0.02986067	2	C05130<br>C00025	1	C05565
chx04723	Retrograde endocannabinoid signaling	0.03018785	1	C00025	1	C00157
chx00380	Tryptophan metabolism	0.03121278	1	C02700	3	C00328<br>C00954<br>C05656

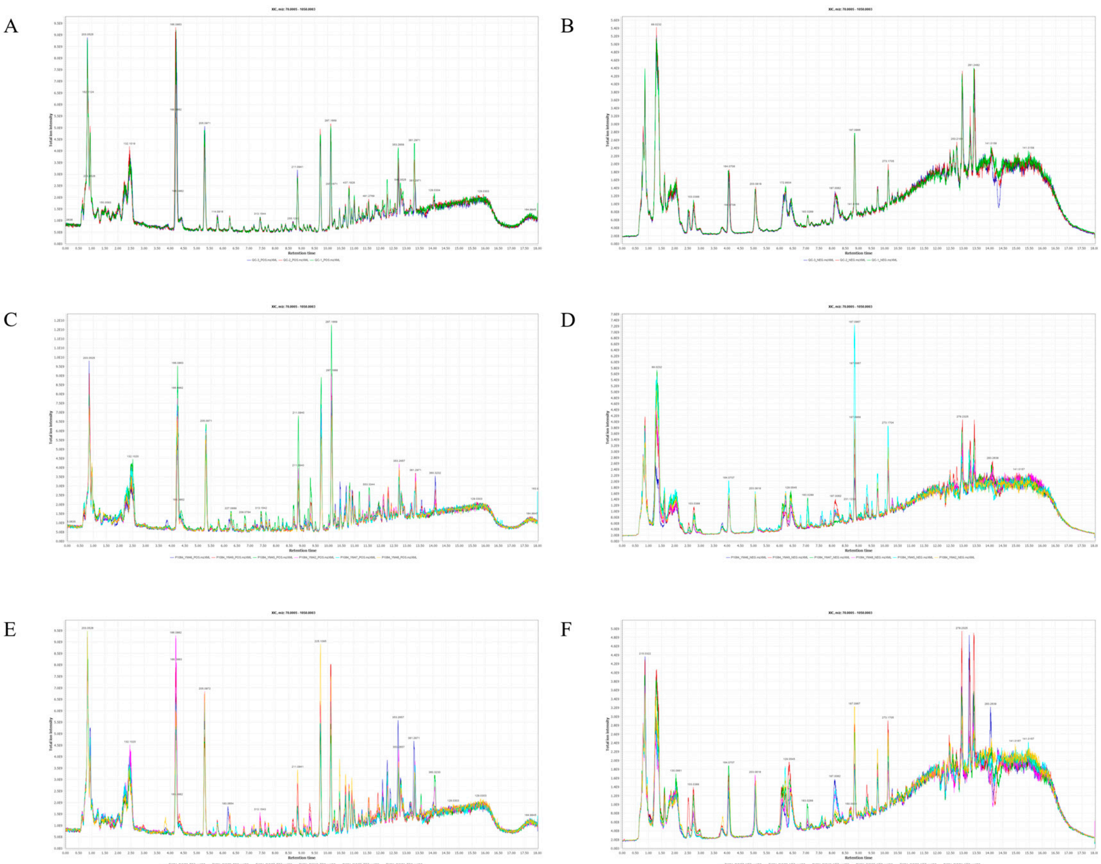

Figure S1. Comparison of TIC of each sample. A, B, Overlap map of QC samples in positive and negative ion mode. C, D, Overlap map of Mo group samples in positive and negative ion mode. E, F, Overlap map of CON group samples in positive and negative ion mode.

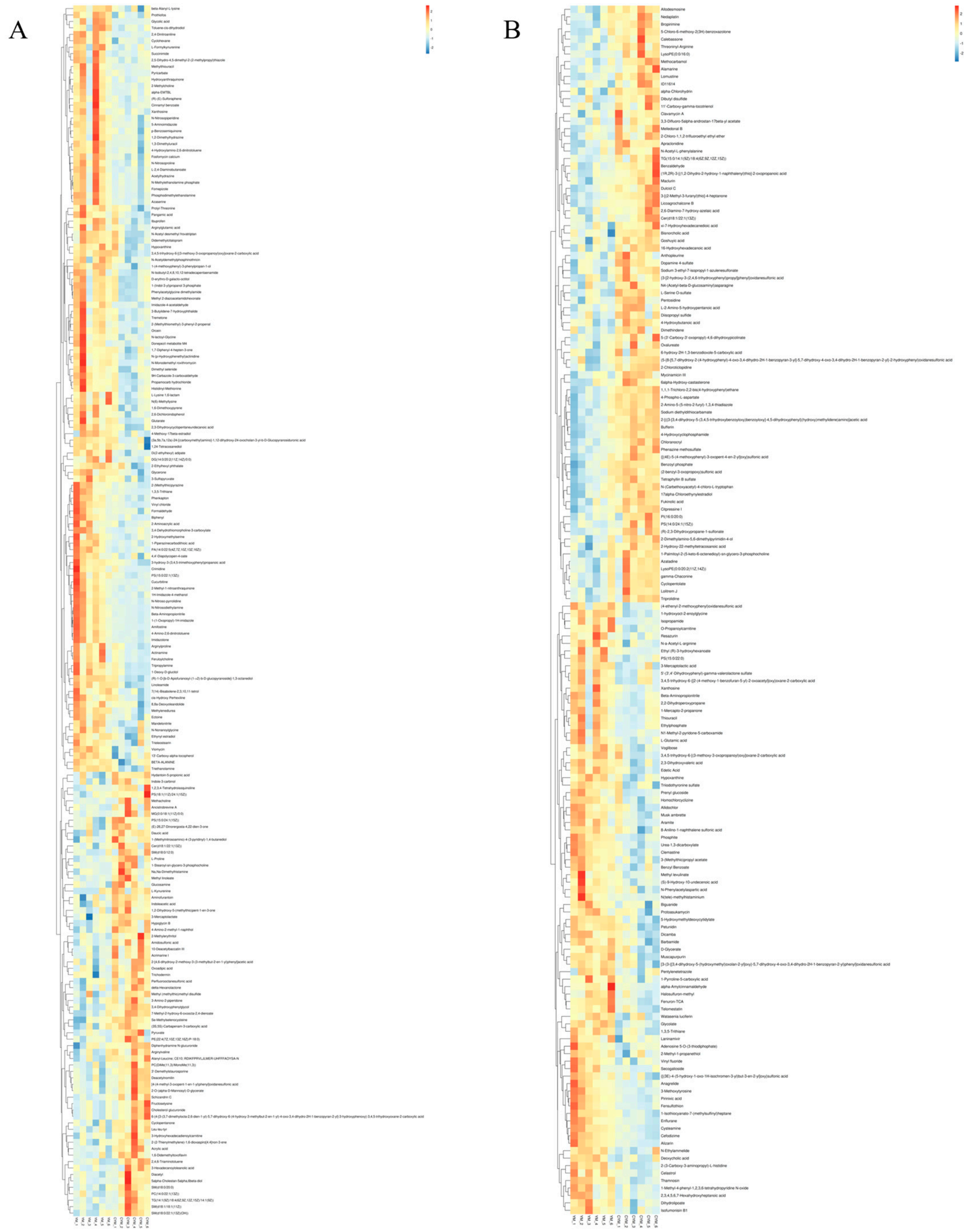

Figure S2. Cluster analysis of DEMs in goat pancreas (A: positive ion model; B: negative ion model).
